# Teachers’ Well-Being, Emotions, and Motivation During Emergency Remote Teaching Due to COVID-19

**DOI:** 10.3389/fpsyg.2022.826828

**Published:** 2022-03-28

**Authors:** Ernesto Panadero, Juan Fraile, Leire Pinedo, Carlos Rodríguez-Hernández, Eneko Balerdi, Fernando Díez

**Affiliations:** ^1^University of Deusto, Bilbao, Spain; ^2^IKERBASQUE Basque Foundation for Science, Bilbao, Spain; ^3^Universidad Francisco de Vitoria, Pozuelo de Alarcón, Spain; ^4^Institute for the Future of Education, Tecnológico de Monterrey, Monterrey, Mexico

**Keywords:** teachers’ well-being, teachers’ emotional reactions, teachers’ motivations, COVID-19, emergency remote teaching

## Abstract

This study explores the effects of the shift to emergency remote teaching (ERT) on teachers’ levels of well-being, emotions, and motivation. A total of 936 Spanish teachers participated in this nationwide survey from all educational levels, thus allowing comparison among levels, which is a novelty and strength of our study. Four aspects were explored: (1) instructional adaptation to ERT; (2) well-being changes and the main challenges in this regard; (3) changes in emotions; and (4) changes in motivation and the main factors. Importantly, we explored a number of teacher characteristics (e.g., gender, age) for the three last aspects. Our results show that teachers felt the impact of ERT on their well-being, emotions, and motivation. Additionally, female teachers, teachers with students of low socioeconomic status (SES), in public schools, and primary and secondary teachers were the most affected groups. This indicates that the impact of ERT differed and some populations of teachers are more at risk of suffering burnout because of ERT.

## Introduction

Principals and teachers are crucial actors in our educational systems and, therefore, they are themselves a form of social capital (Beausaert et al., 2021). Significant attention in educational research has been paid to teachers’ professional development in service (Postholm, 2012) as well as during pre-service teacher training (Aypay, 2009), as it is agreed that teachers’ knowledge about their profession is key for enhancing students’ success within educational systems. Importantly, we also know that as much as teachers need to have technical knowledge, they also need to feel motivated to perform the challenging task of being in front of their students, who have all types of needs. A significant line of research has thus focused on teachers’ well-being, emotions, and motivational levels due to the huge influence they have on the students’ academic success (Sutton and Wheatley, 2003).

The recent COVID-19 pandemic has forced governments in most countries to establish lockdowns. Regular classroom settings were interrupted by these lockdowns and instruction shifted to what has been called ‘emergency remote teaching’ (ERT) (Hodges et al., 2020). The sudden implementation of online teaching worldwide has changed the way teachers and learners communicate and interact, influencing crucial instructional aspects (e.g., assessment practices) (Bozkurt et al., 2020; Rapanta et al., 2020; Tejedor et al., 2021). Unfortunately, most of the teachers were not trained for immersion in online teaching, and these changes seem to have increased the already high levels of stress and demotivation among teachers (Ozamiz-Etxebarria et al., 2021), thus having an impact on the instructional setting and students’ learning. We therefore investigated the gravity of these changes and analyzed how teacher characteristics might have exacerbated or mitigated these negative effects on teachers’ well-being, emotions, and motivation. An important novelty of our study is the comparison among teachers from different educational levels within the same study.

### Teachers’ Well-Being

Defining well-being is not easy as there are many perspectives and theories that try to delimitate this concept. As a general definition, it can be said that well-being is the state of being comfortable, healthy, or happy. Importantly, well-being is not the absolute lack of challenges but “a state…in which every individual realizes his or her own potential, can cope with the normal stresses of life, can work productively and fruitfully, and is able to make a contribution to her or his community” (World Health Organization, 2015, as cited in Beausaert et al., 2021, p. 3). More specifically, Juniper (2011) has defined work-related well-being as “that part of an employee’s overall well-being that they perceive to be determined primarily by work and can be influenced by workplace interventions” (p. 347). When we use well-being in reference to teachers, we are referring to their work at their educational institutions. Teachers’ well-being is influenced by a myriad of contextual factors, such as institutional resources and support (Kumpikaitė-Valiūnienė et al., 2021), workload, or students’ behavior in the classroom (Chan et al., 2021), as well as by teacher-specific personal variables such as personality or engagement at work (Jelińska and Paradowski, 2021).

Teachers’ well-being has been shown to be an important predictor of burnout (Bermejo-Toro et al., 2016), to have a strong relationship with teachers’ motivation and self-efficacy (Collie et al., 2015), and even to influence students’ academic performance (Marks and Louis, 1997). The imposition of ERT created both contextual pressures (e.g., teaching in a completely different instructional environment) and personal constraints (e.g., screen fatigue, psychological challenges, and the added stress of taking care of their children at home while working) (Clark et al., 2021). Additionally, some teachers had to use multiple online platforms in parallel including learning management systems (Moodle or E-class) and communicative platforms (Google Meet, Zoom or MS Teams), which complicates the organization of tasks (Kanetaki et al., 2021). In this line, teachers have also increased the use of gamified activities that involve coping with technical difficulties, although they facilitate students’ motivation given that they contribute fun to learning (Krouska et al., 2022). We therefore explored the variation in teachers’ well-being before and after ERT, analyzed the main reasons for these changes, and sought to uncover how teachers dealt with these challenges. This information can be used for more tailored and specific interventions.

### Teachers’ Emotions

Obviously, there is a direct link between teachers’ well-being and the type of emotions they experience at work (Day and Qing, 2009). While teachers’ emotions have received less attention than students,’ there a significant body of knowledge has been produced around this topic (Frenzel, 2014). Without a doubt, teachers’ emotions are important on their own: no one wants teachers to suffer from burnout or depression; nevertheless, their emotions are also crucial for students’ academic achievement. Frenzel et al. (2021) developed a model in which teachers’ emotions affect students *via* three teaching behaviors: relationship building, non-verbal social messages, and instructional strategies. They also established a direct transmission effect between teachers’ emotions and student outcomes (i.e., students’ emotions, beliefs, motivation, discipline, and performance), which is also supported by previous research (Sutton and Wheatley, 2003).

Importantly, teachers have a higher risk of burnout than other professions (Hakanen et al., 2006), and this negative risk is strongly influenced by the negative emotions that professionals feel at their workplace (Chang, 2009). Currently, teacher dropout rates are fairly high owing to psychological causes related to their experience of negative emotions (e.g., sadness, tiredness, or anxiety disorders) (Frenzel, 2014). In this vein, teachers have managed potential intrapersonal conflicts during the pandemic, as the job requires numerous social contacts, and social distancing might be difficult to maintain, which might have produced negative emotions (Nabe-Nielsen et al., 2021). It is thus important to identify what strategies are helping teachers cope with stressful situations, as the increased use of avoidance coping is associated with increasing levels of stress and a variety of negative emotions (e.g., anxiety, anger, sadness, and loneliness) (MacIntyre et al., 2020). Considering the above, exploring teachers’ emotions in the context of ERT is key to our understanding of how they have coped and what impact this situation has had on teachers.

### Teachers’ Motivation

As discussed in an empirical review by Sutton and Wheatley (2003), teachers’ emotions have a direct reciprocal influence on their motivation. According to Watt and Richardson (2015), research on teachers’ motivation has received a significant impulse focusing around three main motivation theories: expectancy-value, achievement goal, and self-determination. Importantly, other authors have claimed that teachers’ motivation cannot be explained based on the same models we hold for students, as their achievement context is different (Fives and Buehl, 2016). Regardless of these foundational arguments, it is without a doubt agreed that teachers’ motivation is key to their social capital within educational systems (Han and Yin, 2016; Beausaert et al., 2021).

As with teachers’ emotions, motivation also affects the type of instructional strategies teachers employ (Fives and Buehl, 2016) and students’ outcomes, such as help seeking or cheating (Butler and Shibaz, 2008). It is important to consider the main factors influencing teachers’ motivation, and much research has considered how contextual factors influence teachers’ motivation (Fives and Buehl, 2016). Aspects such as institutional climate, sense of belonging to the community, or relationships with students largely influence teachers’ motivation.

As with well-being and emotions, it is to be expected that the impact of ERT has affected teachers’ motivation due to the constraints and pressures of the exceptional situation; interestingly, this has received less attention than well-being. Next we briefly outline three studies on the topic. Kulikowski et al. (2021a) found four core job characteristics (task identity, task significance, autonomy, and social dimension) that might decrease as a result of ERT, thus affecting teachers’ motivation and job performance. Khanal et al. (2021) found that teachers in private schools reported being intrinsically and extrinsically demotivated due to several factors, such as heavy workload, students’ disruptive behaviors, and lack of professional development events, among others (Khanal et al., 2021). Finally, in the same direction, the findings of Panisoara et al. (2020) showed that, during remote teaching, teachers’ extrinsic motivation significantly increased occupational stress (i.e., burnout) whereas intrinsic motivation decreased it. Due to these previous results it is therefore important to explore teachers’ motivational levels, the main factors influencing their motivation, and what strategies they use to regulate their motivation.

### Aim and Research Questions

As discussed above, there are direct links between teachers’ well-being, emotions, and motivational level (e.g., Sutton and Wheatley, 2003; Fives and Buehl, 2016; Frenzel et al., 2021). These elements are so interrelated that they depend on each other and, at the same time, they are also identifiable as independent constructs with large amount of empirical evidence behind each of them. We therefore decided to explore them to gain a comprehensive picture of the effects of ERT while using independent questions. We also investigated if teachers had changed their instructional settings, as these changes would imply effort and a significant amount of time, therefore also impacting their well-being, emotions, and motivation. Our aim was to investigate how ERT affected the well-being, emotional state, and motivation of teachers by exploring the changes, challenges, and strategies used to cope, while exploring whether the teachers’ characteristics might have exacerbated or mitigated these effects. The study is organized around four research questions (RQ):

RQ1: Did teachers receive training for the ERT, did they change their instructional setting, and did teachers’ characteristics influence these changes?
RQ2: Did teachers’ well-being change, what were the challenges to well-being, and what characteristics influenced this change?
RQ3: Did teachers’ emotions change, and what characteristics influenced this change?
RQ4: What was the teachers’ motivational level, which factors affected it, and what characteristics influenced this level?

## Materials and Methods

### Participants

The sample included 936 Spanish teachers from early childhood education (*n* = 64; 6.8%), primary education (*n* = 207; 22.1%), secondary education (*n* = 337; 36%), vocational education (*n* = 85; 9.1%), higher education (*n* = 192; 20.5%), and other educational contexts (*n* = 51; 5.4%). In terms of gender distribution, 641 (68.5%) of the participants were female. Of the sample, 798 (85.3%) worked in public institutions, 90 (9.6%) in state-subsidized institutions, and 48 (5.1%) in private institutions. The average age of participants was 44.8 years (*SD* = 10.88), and they had 15.8 years of teaching experience (*SD* = 10.66). Regarding qualifications, the entire sample held a university degree, 160 (17.1%) had a master’s degree, and 190 (20.3%) had a PhD. All 17 autonomous communities of Spain were represented. We used convenience sampling of those teachers who voluntarily decided to participate. We sent a summary of the results to participants who provided an email.

### Instrument

Our self-report survey contained 91 questions. First, we asked for demographic and personal data, including gender; age; location; educational level; school type (public, state-subsidized, or private); qualifications; years of teaching experience; and if they had to cease working due to COVID-19. We then asked participants about their area of teaching expertise, the theoretical/practical character of the topic they teach, the availability of technical equipment, the socioeconomic profile of their students, and their assessment practices. Finally, we asked questions regarding instructional teaching changes, teachers’ perceived well-being, teachers’ positive and negative emotions, and teachers’ level of motivation.

### COVID-19 in Spain

Between March 9, 2020 (beginning in Madrid and the Basque Country) and March 16, 2020, all educational institutions at all levels were gradually closed. The state of alarm ended on June 21, 2020, with some restrictions remaining.

### Procedure

We developed the survey on an online platform and disseminated it by email, text messages, and social network sites. For email distribution, we used a database from a previous research project to reach over 8,000 teachers at all educational levels. We also asked the participants to share the information with their colleagues. Although the Spanish government declared the lockdown in March 2020, including the closure of educational institutions, we waited until April to distribute the instrument. The rationale was to wait some weeks for teachers to have a more precise and extended experience of what ERT entailed, including the regulatory actions and guidelines released by the educational administration.

### Data Analysis

We conducted several statistical analyses to investigate the effects of the ERT on teachers’ well-being, emotion, and motivation in comparison with different teachers’ characteristics. We therefore calculated contingency tables and Chi-squared tests to answer RQ1 and RQ3. We used a Wilcoxon signed-rank test for RQ2. Finally, one-way ANOVAs were performed to answer RQ4.

## Results

### RQ1: Did Teachers Receive Training for the Emergency Remote Teaching, Did They Change Their Instructional Setting, and Did Teachers’ Characteristics Influence These Changes?

**FIGURE 1:**
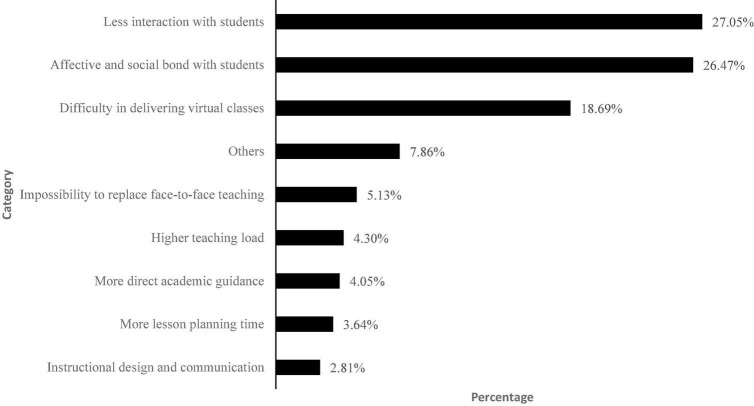
Main changes in teaching practices (*n* = 1209). *Less interaction with students* includes less feedback, efficiency and dynamism with families and students, and no motivation (12.49%), difficulty for checking students’ progress (7.86%), and difficulty for contacting students’ families (6.7%). *Difficulty in delivering virtual classes* includes difficulty in proposing practices and explaining the subject (11%), and ICT dependency (7.69%). *Higher teacher load* refers to poor work-life balance, less privacy, lack of specific work schedule (reply to emails outside of working hours). *Others* includes categories with a presence lower than 2%: reaching students with different needs (1.99%), responding to students’ self-learning (1.41%), responding to students’ questions in class (1.08%), more flexible schedules (0.91%), opportunity to innovate in the subject (0.83%), more feedback (0.74%), no changes (0.58%), and unclassifiable answer (0.32%).

We first asked teachers about their training for ERT: 68.23% did not receive training, while 31.77% had received it. The ones who had received it considered the training as: Excellent (10.06%), Satisfactory (38.25%), Sufficient (28.52%), Insufficient (20.13%), and Poor (1.67%). Additionally, we asked in an open-ended question what the main changes in their teaching practice had been. As shown in Figure 1, these were related to less interaction with their students (27.05%), affective and social bound with of students (26.47%), and in delivering virtual classes, proposing practices, and explaining the subject (18.69%).

**FIGURE 2:**
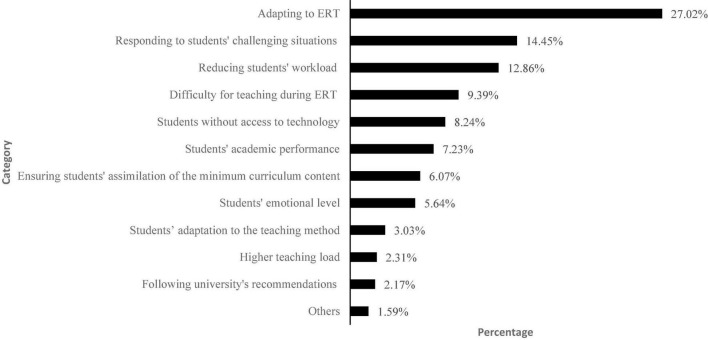
Main reasons for introducing instructional changes (*n* = 692). *Responding to students’ challenging situations* includes problematic circumstances for students (7.08%), students’ autonomy when studying for the subject (4.48%), and students’ difficulty in understanding the subject and submitting assignments (2.89%). *Reducing students’ workload* refers to avoiding students being overwhelmed or responding to families’ requests to reduce students’ workload.

Finally, we asked about the reasons for those instructional changes (Figure 2), with the following being the main ones: adapting to ERT (27.02%), responding to students’ challenging situations (14.45%), and reducing students’ workload (12.86%).

We subsequently determined the relationship between teacher characteristics and instructional changes. As shown in Table 1, there was a significant relationship between educational level, gender, school type, students’ socioeconomic status (SES), and changes in course objectives and contents. Educational level was more strongly related to changes in course objectives and content (largest Cramer’s V). Conversely, there was not a significant relationship between age and years of experience and changes in course objectives and contents. Consequently, teachers who were more likely to pose less challenging course objectives and content were those working in secondary education, or whose students came from low SES conditions. Contrary, higher education teachers or those with intermediate-low SES students were more likely to pose course objectives and contents as challenging as before ERT. Remarkably, female teachers or public-school teachers were more likely to pose the same or less challenging course objectives and content.

**TABLE 1:** Changes in course objectives and contents.

	*N*	*More challenging*	*Same*	*Less challenging*	*Other*	*Chi-squared test*	
*Total*	936	15	342	512	67		
***Age***							
Less than 37	242	4	81	138	19	Cramer’s V	0.07
Between 38 and 45	237	4	80	140	13	*X*^2^ (9, N = 935)	13.29
Between 46 and 54	249	4	85	140	20	Significance level	0.15
More than 55	207	2	96	94	15		
***Educational level***							
Early childhood	64	1	15	39	9	Cramer’s V	0.22
Primary education	207	3	36	157	11	*X*^2^ (15, N = 936)	137.07
Secondary education	337	3	108	198	28	Significance level	<0.001
Higher education	192	4	130	50	8		
Vocational education	85	3	32	44	6		
Other level	51	1	21	24	5		
***Experience years***							
Less than 6	212	2	71	122	17	Cramer’s V	0.07
Between 6 and 15	211	3	51	144	13	*X*^2^ (9, N = 789)	10.24
Between 16 and 24	176	3	61	95	17	Significance level	0.33
More than 25	190	2	61	113	14		
***Gender***							
Female	641	7	216	368	50	Cramer’s V	0.11
Male	295	8	126	144	17	*X*^2^ (3, N = 936)	11.70
						Significance level	0.01
***School type***							
State-subsidized & private	138	1	79	50	8	Cramer’s V	0.18
Public	798	14	263	462	59	*X*^2^ (3, N = 936)	30.29
						Significance level	<0.001
***Students’ Socioeconomic Status***							
Low	267	7	70	176	14	Cramer’s V	0.11
Intermediate low	291	5	107	152	27	*X*^2^ (9, N = 921)	35.26
Intermediate high	197	1	75	106	15	Significance level	<0.001
High	166	2	84	69	11		

Next, we investigated the relationship between several teacher characteristics and changes in student workload. As shown in Table 2, there was a significant relationship between age, educational level, experience years, gender, school type, students’ SES, and changes in student workload. Educational level was also more strongly related to the changes in student workload (largest Cramer’s V). Teachers were more likely to provide a reduced workload if they were younger than 37 years old, worked in secondary education, had 6–15 years of experience, or had low SES students. In contrast, teachers were more likely to provide the same workload to their students if they worked in higher education, were older, most experienced, or had intermediate-low SES students. Female teachers and public-school teachers were more likely to provide the same or a reduced workload to students.

**TABLE 2:** Changes in students’ workload.

	N	More workload	Same	Less workload	Other	Chi-squared test	
Total	936	40	321	533	42		
***Age***							
Less than 37	242	13	62	160	7	Cramer’s V	0.12
Between 38 and 45	237	7	72	149	9	*X*^2^ (9, N = 935)	39.08
Between 46 and 54	249	7	88	138	16	Significance level	<0.001
More than 55	207	12	99	86	10		
***Educational level***							
Early childhood	64	1	8	46	9	Cramer’s V	0.26
Primary education	207	2	29	167	9	*X*^2^ (15, N = 936)	187.38
Secondary education	337	15	98	215	9	Significance level	<0.001
Higher education	192	14	123	46	9		
Vocational education	85	6	37	39	3		
Other level	51	2	26	20	3		
***Experience years***							
Less than 6	212	11	59	135	7	Cramer’s V	0.09
Between 6 and 15	211	6	50	150	5	*X*^2^ (9, N = 789)	18.19
Between 16 and 24	176	3	53	107	13	Significance level	0.03
More than 25	190	5	66	109	10		
***Gender***							
Female	641	28	197	383	33	Cramer’s V	0.12
Male	295	12	124	150	9	*X*^2^ (3, N = 936)	12.36
						Significance level	0.01
***School type***							
State-subsidized & private	138	7	66	60	5	Cramer’s V	0.12
Public	798	33	255	473	37	*X*^2^ (3, N = 936)	14.31
						Significance level	0.00
***Students’ Socioeconomic Status***							
Low	266	8	67	180	11	Cramer’s V	0.11
Intermediate low	291	10	107	157	17	*X*^2^ (9, N = 919)	34.30
Intermediate high	197	13	64	114	6	Significance level	<0.001
High	165	9	79	71	6		

### RQ2: Did Teachers’ Well-Being Change, What Were the Challenges to Well-Being, and What Characteristics Influenced This Change?

We asked teachers about their well-being before and during ERT using a nine-point continuous scale (from very low to very high). We then compared both levels of perceived well-being in relation to teacher characteristics (Table 3). In this case, a non-parametric test (Wilcoxon rank-test) was chosen to determine whether the changes in teachers’ perceived well-being were statistically significant, as the data did not show a normal distribution.

**TABLE 3:** Teachers’ well-being before and after emergency remote teaching.

	*N*	Mean before	Mean after	Negative ranks	Positive ranks	Ties	Wilcoxon rank test	Significance level
Total	936	7.06 (2.07)	5.22 (2.21)	639	93	204	*Z* = −19.59	<0.001
***Age***								
Less than 37	242	7.14 (2.01)	5.42 (2.05)	163	30	49	*Z* = −9.57	<0.001
Between 38 and 45	237	7 (2.09)	4.94 (2.27)	161	22	54	*Z* = −10.39	<0.001
Between 46 and 54	249	7 (2.08)	5.12 (2.17)	183	21	45	*Z* = −10.23	<0.001
More than 55	207	7.16 (2.07)	5.43 (2.32)	132	20	55	*Z* = −8.97	<0.001
***Educational level***								
Early childhood	64	7.23 (2.08)	5.01 (2.28)	42	9	13	*Z* = −5.30	<0.001
Primary education	207	7.17 (1.99)	4.85 (2.18)	155	17	35	*Z* = −9.94	<0.001
Secondary education	337	7.08 (1.85)	5.36 (1.92)	237	43	57	*Z* = −11.35	<0.001
Higher education	192	6.69 (2.66)	5.26 (2.66)	112	13	67	*Z* = −8.39	<0.001
Vocational education	85	7.35 (1.65)	5.42 (2.09)	61	5	19	*Z* = −6.46	<0.001
Other level	51	7.19 (1.83)	5.52 (2.23)	32	6	13	*Z* = −4.21	<0.001
***Experience years***								
Less than 6	212	7.15 (1.9)	5.53 (2.02)	143	27	42	*Z* = −8.65	<0.001
Between 6 and 15	211	7.15 (1.86)	5.06 (2.02)	157	20	34	*Z* = −10.08	<0.001
Between 16 and 24	176	7.31 (1.82)	4.93 (2.19)	131	14	31	*Z* = −9.51	<0.001
More than 25	190	7.21 (1.75)	5.52 (2.01)	125	24	41	*Z* = −8.15	<0.001
***Gender***								
Female	641	7.09 (2.06)	5.08 (2.15)	459	61	121	*Z* = −16.57	<0.001
Male	295	7.02 (2.1)	5.51 (2.31)	180	32	83	*Z* = −10.36	<0.001
***School type***								
State-subsidized & Private	138	7.05 (1.89)	5.67 (1.97)	84	18	36	*Z* = −6.73	<0.001
Public	798	7.07 (2.11)	5.14 (2.24)	555	75	168	*Z* = −18.38	<0.001
***Students’ Socioeconomic Status***								
Low	266	6.83 (2.21)	4.83 (2.14)	184	32	50	*Z* = −10.31	<0.001
Intermediate low	291	6.94 (2.24)	5.06 (2.35)	189	23	79	*Z* = −11.00	<0.001
Intermediate high	197	7.31 (1.78)	5.7 (2)	134	19	44	*Z* = −8.98	<0.001
High	165	7.36 (1.84)	5.47 (2.2)	120	15	30	*Z* = −8.54	<0.001

As indicated by the negative ranks in Table 3, most of the teachers declared that their perceived well-being diminished after ERT, and this decrease was significant (*Z* = −19.59, *p* < 0.001). A similar result was also identified for each of the teacher characteristics analyzed, as confirmed by the larger negative ranks and the statistically significant Wilcoxon rank tests shown in Table 3. As such, the teachers who reported the lowest perceived well-being were those aged between 38 and 45 years (*M* = 4.94, *SD* = 2.27), who worked in primary education (*M* = 4.85, *SD* = 2.18), whose years of experience were between 16 and 24 years (*M* = 4.93, *SD* = 2.19), who were female (*M* = 5.08, *SD* = 2.15), who taught in public schools (*M* = 5.14, *SD* = 2.24), or whose students came from low SES (*M* = 4.83, *SD* = 2.14).

**FIGURE 3:**
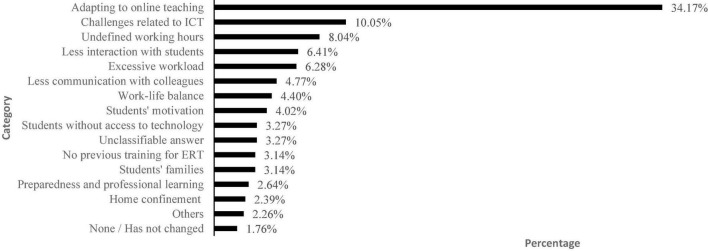
Main challenges for teachers’ well-being (*n* = 796). *Adapting to online teaching* includes reaching all students and teaching them efficiently (9.3%), remote teaching (5.15%), academic guidance and support (3.27%), lesson planning (2.39%), no teaching material (2.26%), coping uncertainty and stress (2.01%), providing/receiving feedback (2.01%), assessment (1.88%), working remotely (1.63%), excessive bureaucracy and paperwork (1.63%), nonconformity with lessons delivery (1.51%), and receiving students’ low-quality work (1.13%). *Students’ families* includes conflict with families (1.26%), criticism from families (1.01%), and adapting to families without resources (0.87%). *Home confinement* refers to not leaving home and isolation.

We also asked teachers about the main challenges to their well-being during ERT. According to Figure 3, the main challenges were related to adapting to online teaching (34.17%), information and communication technologies (ICT) (10.05%), and the undefined working hours that blurred the boundaries between working hours and personal time (8.04%).

### RQ3: Did Teachers’ Emotions Change, and What Characteristics Influenced This Change?

We asked our participants whether their emotions had changed during ERT using four comparative options: more, same, less, and never have this feeling. Table 4 displays their answers. Apparently, positive emotions decreased, as teachers reported lower levels of happiness (50%), hope (35.04%), and relief (45.52%). Conversely, there was an increase in negative emotions: more anxiety (60.92%), nervousness (59.06%), and sadness (43.23%). We then explored the relationship between several teacher characteristics and changes in their positive and negative emotions. The complete contingency tables for this analysis are available upon request, but the main results are summarized in Table 5.

**TABLE 4:** Changes in teachers’ emotions during emergency remote teaching.

	More		Same		Less		Never have this feeling	
				
*Positive emotions* (*n* = 916)	*N*	Percentage	*N*	Percentage	*N*	Percentage	*N*	Percentage
Happiness	44	4.8%	402	43.89%	458	50%	12	1.31%
Hope	177	19.32%	400	43.67%	321	35.04%	18	1.97%
Pride	279	30.46%	441	48.14%	145	15.83%	51	5.57%
Relief	67	7.31%	344	37.55%	417	45.52%	88	9.61%
***Negative emotions (*n* = 916)***								
Anxiety	558	60.92%	225	24.56%	79	8.62%	54	5.9%
Nervousness	541	59.06%	255	27.84%	79	8.62%	41	4.48%
Shame	70	7.64%	412	44.98%	121	13.21%	313	34.17%
Sadness	396	43.23%	316	34.50%	93	10.15%	111	12.12%
Boredom	204	22.27%	264	28.82%	184	20.09%	264	28.82%

**TABLE 5:** Teachers’ positive and negative emotions during emergency remote teaching.

		Positive emotions				Negative emotions				
			
		Happiness	Hope	Pride	Relief	Anxiety	Nervousness	Shame	Sadness	Boredom
Age	Cramer’s V	0.08	0.07	0.07	0.08	0.08	0.08	0.05	0.12	0.1
	*X*^2^ (9, *N* = 916)	16.88	12.54	11.86	16.4	18.84	17.56	7.67	38.69	25.34
	Significance level	0.051	0.19	0.22	0.06	0.03	0.04	0.57	<0.001	0.00
	Interpretation	N.A.	N.A.	N.A.	N.A.	Teachers aged between 38 and 45 years are more likely to feel more anxious	Teachers aged 37 years or less are more likely to feel more nervous	N.A.	Teachers aged 37 years or less are more likely to feel sadder	Teachers aged 37 years or less are more likely to feel more bored
Educational level	Cramer’s V	0.11	0.06	0.07	0.12	0.12	0.12	0.16	0.13	0.15
	*X*^2^(15, *N* = 916)	34.78	8.57	14.84	37.62	36.91	40.98	67.68	46.76	57.86
	Significance level	0.03	0.9	0.46	0.00	0.00	<0.001	<0.001	<0.001	<0.001
	Interpretation	Primary and secondary teachers are more likely to feel less happy	N.A.	N.A.	Primary and secondary teachers are more likely to feel less relieved	Primary and secondary teachers are more likely to feel anxious	Primary and secondary teachers are more likely to feel nervous	Secondary teachers are more likely to never feel ashamed	Primary and secondary teachers are more likely to feel sad	Secondary teachers are more likely to never feel bored
Experience years	Cramer’s V	0.11	0.05	0.08	0.05	0.07	0.08	0.05	0.1	0.11
	*X*^2^ (9, *N* = 789)	27.94	4.88	15.44	5.70	10.70	16.38	6.07	22.55	27.02
	Significance level	0.00	0.85	0.08	0.77	0.3	0.06	0.73	0.01	0.00
	Interpretation	Teachers whose experience is less than 6 years are more likely to feel less happy	N.A.	N.A.	N.A.	N.A.	N.A.	N.A.	Teachers whose experience is less than 6 years and between 7 and 15 years are more likely to feel sad	Teachers whose experience is more than 25 years are more likely to never feel bored
Gender	Cramer’s V	0.09	0.05	0.09	0.13	0.14	0.16	0.11	0.16	0.13
	*X*^2^ (3, N = 916)	6.83	2.45	7.23	15.37	16.88	21.89	10.88	22.57	15.10
	Significance level	0.08	0.49	0.07	0.00	0.00	<0.001	0.01	<0.001	0.00
	Interpretation	N.A.	N.A.	N.A.	Women are more likely to feel less relieved	Women are more likely to feel anxious	Women are more likely to feel nervous	Women are more likely to feel similarly ashamed	Women are more likely to feel sad	Women are more likely to never feel bored
School type	Cramer’s V	0.13	0.12	0.12	0.08	0.1	0.09	0.04	0.07	0.03
	*X*^2^ (3, *N* = 916)	15.07	13.91	12.85	6.09	9.60	7.11	1.57	4.16	0.83
	Significance level	0.00	0.00	0.01	0.11	0.02	0.07	0.67	0.25	0.84
	Interpretation	Teachers from public schools are more likely to feel less happy	Teachers from public schools are more likely to feel similarly hopeful	Teachers from public schools are more likely to feel similarly proud	N.A.	Teachers from public schools are more likely to feel anxious	N.A.	N.A.	N.A.	N.A.
Students’ Socioeconomic Status	Cramer’s V	0.09	0.08	0.09	0.08	0.06	0.07	0.07	0.1	0.06
	*X*^2^ (9, *N* = 899)	23.11	18.07	20.65	16.79	8.07	11.53	13.42	26.06	8.35
	Significance level	0.01	0.03	0.01	0.052	0.53	0.24	0.15	0.00	0.5
	Interpretation	Teachers whose students come from low SES are more likely to feel less happy	Teachers whose students come from intermediate low SES are more likely to feel similarly hopeful	Teachers whose students come from intermediate low SES are more likely to feel similarly proud	N.A.	N.A.	N.A.	N.A.	Teachers whose students come from low SES are more likely to feel sad	N.A.

Age was not significantly related to any positive emotion. In terms of the educational level, primary and secondary teachers were more likely to feel less happy and less relieved. Likewise, teachers with less than 6 years’ experience were more likely to feel less happy. Women were more likely to feel less relieved than men. Teachers from public schools were more likely to feel less happy, similarly hopeful, and similarly proud. Finally, teachers whose students come from low SES were more likely to feel less happy, while teachers whose students came from low-intermediate SES were more likely to feel similarly hopeful and proud.

Regarding negative emotions, age was significantly related to anxiety, nervousness, sadness, and boredom. Teachers aged 37 years or less were more likely to feel more nervous, sad, and bored. In addition, respondents aged between 38 and 45 years were more likely to feel more anxious. In terms of educational level, primary and secondary teachers were more anxious, nervous, sad, and bored. In relation to years of experience, teachers with less than 15 years’ experience were more likely to feel sad, while most experienced teachers were more likely never to feel bored. Female teachers were more likely to feel anxious, nervous, and sad. Public school teachers were more likely to feel anxious. Finally, teachers with low SES students were more likely to feel sad.

### RQ4: What Was the Teachers’ Motivational Level, Which Factors Affected It, and What Characteristics Influenced This Level?

**FIGURE 4:**
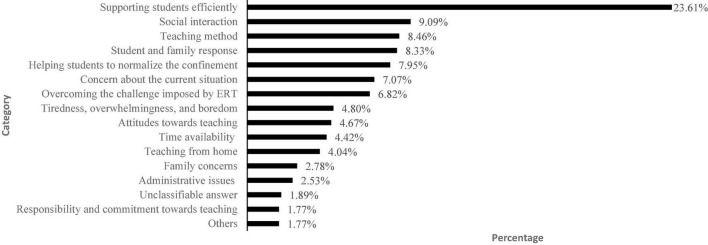
Main factors influencing teachers’ motivation levels (*n* = 792). *Social interaction* includes social contact (4.67%), isolation/confinement (1.64%), communication (1.39%), and interaction with colleagues (1.39%). *Teaching method* includes feelings about teaching (2.27%), impossibility to provide immediate feedback and appropriate guidance (1.77%), technological resources availability (1.52%), achieving teaching objectives (1.52%), and no preparation for facing ERT (1.38%).

We also asked teachers about their work motivation during ERT using a nine-point continuous scale (from very low to very high). The average teachers’ motivation level was 5.68 (*SD* = 2.05). We then asked teachers about the factors influencing their level of motivation (Figure 4) and found that most reported factors were related to supporting students efficiently (23.61%), social interaction (9.09%), and teaching method (8.46%).

Subsequently, we determined whether teachers’ work motivation differed across several of their characteristics through one-way ANOVA. Although the variable “work motivation” did not follow a normal distribution, the one-way ANOVA is robust against this assumption (Blanca et al., 2017). Levene’s statistic was significant in all cases, thus fulfilling the assumption of homogeneity of variance. These results are provided in Table 6.

**TABLE 6:** ANOVA results for teachers’ motivation during emergency remote teaching.

	*N*	Mean		Sum of squares	df	Mean square	*F*	Significance level
***Age***								
Less than 37	241	5.60 (2.06)	Between groups	18.87	3	6.29	1.52	0.21
Between 38 and 45	234	5.53 (2.06)	Within groups	3802.45	916	4.15		
Between 46 and 54	245	5.69 (2.07)	Total	3821.32	919			
More than 55	200	5.93 (1.91)						
***Educational level***								
Early childhood	64	5.54 (2.02)	Between groups	67.24	5	13.45	3.25	0.01
Primary education	207	5.63 (2.09)	Within groups	3786.34	915	4.14		
Secondary education	336	5.8 (2.03)	Total	3853.58	920			
Higher education	178	5.21 (1.95)						
Vocational education	85	6.12 (2.02)						
Other level	51	6.01 (2.09)						
***Experience years***								
Less than 6	212	5.74 (2.01)	Between groups	31.66	3	10.55	2.54	0.06
Between 6 and 15	211	5.56 (2.03)	Within groups	3258.56	785	4.15		
Between 16 and 24	176	5.75 (2.1)	Total	3290.23	788			
More than 25	190	6.11 (2)						
***Gender***								
Female	632	5.57 (2.06)	Between groups	20.34	1	20.34	4.88	0.03
Male	289	5.89 (2)	Within groups	3833.24	919	4.17		
			Total	3853.58	920			
***School type***								
State-subsidized & Private	138	5.76 (1.91)	Between groups	1.16	1	1.16	0.28	0.6
Public	783	5.66 (2.07)	Within groups	3852.42	919	4.19		
			Total	3853.58	920			
***Students’ Socioeconomic Status***								
Low	264	5.45 (2.07)	Between groups	61.31	3	20.44	4.94	0.00
Intermediate low	281	5.54 (2.05)	Within groups	3722.37	900	4.14		
Intermediate high	195	6.14 (1.87)	Total	3783.68	903			
High	164	5.7 (2.1)						

First, statistically significant differences were found in teachers’ work motivation across educational level [*F*(5,915) = 3.25, *p* = 0.01]. A Tukey HSD *post-hoc* test revealed that the work motivation of higher education teachers (*M* = 5.21, *SD* = 1.95) was statistically significantly lower than the work motivation of secondary education (*M* = 5.8, *SD* = 2.203, *p* = 0.03) and vocational education (*M* = 6.12, *SD* = 2.02, *p* = 0.01) teachers.

Second, there were statistically significant differences in teachers’ work motivation across gender [*F*(1,919) = 4.88, *p* = 0.027], with female teachers (*M* = 5.57, *SD* = 2.06) being less motivated about their work than male teachers (*M* = 5.89, *SD* = 2).

Finally, statistically significant differences in teachers’ work motivation were identified across students’ SES [*F*(3,900) = 4.94, *p* = 0.00]. A Tukey HSD *post-hoc* test suggested that the work motivation of teachers whose students come from intermediate-high SES conditions (*M* = 6.14, *SD* = 1.87) was statistically significantly higher than the work motivation of teachers whose students come from intermediate-low (*M* = 5.54, *SD* = 2.05, *p* = 0.00) and low SES conditions (*M* = 5.45, *SD* = 2.07, *p* = 0.00).

## Discussion

We investigated if and how ERT impacted teachers’ well-being (RQ2), emotions (RQ3), and motivation (RQ4), while exploring if teacher characteristics influenced these effects. For a more complete analysis, we further investigated if teachers received training for the new context and if they changed their instructional strategies (RQ1), as these could have an impact on their workload and, therefore, their well-being, emotions, and motivation. Importantly, we do not know of any research in which such level of comparison among educational level and teacher characteristics among COVID-19 publications.

In regard to RQ1, the majority of teachers did not receive specific training for ERT, and half of those who did were not satisfied with it. Our results align with previous research that highlights the lack of preparation and support the teachers had received for providing quality teaching during ERT (Whalen, 2020; Zhang et al., 2020), as well as their lack of preparation for using adaptive learning activities in this new context (Troussas et al., 2021b). Additionally, the main instructional changes reported were less interaction with students and caring for the students affectively and socially. In particular, the teachers reported the need to acquire the pedagogical content knowledge to design and carry out meaningful experiences in a remote setting (Rapanta et al., 2020). Previous research has also found instructional changes, such as the majority of teachers changing or eliminating assignments or exams (de Boer, 2021) or they lowering their expectations regarding students’ work (Johnson et al., 2020). The greatest challenge for university teachers was the importance of establishing affective connections with their students (Alvarez, 2020) as learning is not just about grades, it is also a matter of care and compassion (Bozkurt and Sharma, 2020). In this scenario, it was expected that teachers’ well-being, emotions, and motivation would be affected and that teacher characteristics would mediate these effects.

Regarding how the teachers’ characteristics influenced instructional changes, we found that female teachers, teachers working in secondary education, teachers of low SES students, or in public schools, were those who reported decreasing the instructional goals in their courses; while university teachers and those with students from intermediate-low SES maintained the pre-lockdown levels. Finally, younger teachers (under 37 years), with intermediate levels of experience (6–15 years), working in secondary education, or with students from low SES, reduced their students’ workload. In contrast, the following types maintained the same workload: older teachers, those with more experience, university teachers, or those with students with intermediate-low SES.

Regarding teachers’ well-being (RQ2), the participants massively reported a decrease compared to the previous period, which is in line with previous research (Alves et al., 2020). Levels of well-being were lower among a particular age range (38–45), for primary education teachers, those with longer experience (16–24 years), females, public schools, and students from low SES. Regarding gender differences, family-related time use and caring responsibilities appear to play a role (Etheridge and Spantig, 2020; Klapproth et al., 2020); however, there are some inconsistencies in the scientific literature regarding the relationship between gender and well-being (Alves et al., 2020), because the gender variable is a predictor of professional well-being: sometimes from a positive perspective (female teachers are more satisfied), sometimes from a negative perspective (male teachers are more satisfied) and other times it is not a significant predictor. Some studies have also discussed innovative instructional factors such as reinforcing teacher self-efficacy and, in turn, teacher well-being (Hascher et al., 2021). However, in this study, interestingly, the main challenges were either with instructional factors or technology, showing that teachers struggled to deliver their courses in the new learning environment. This aligns with previous research by Duraku and Hoxha (2020) who found that an insufficient level of skills and knowledge related to the use of technology created anxiety, overload, insecurity, stress, and job dissatisfaction to teachers. One possible intervention suggested by Anderson et al. (2021) is training teachers in a growth mindset as this might be positive for their well-being, which is necessary to offer student-centered learning opportunities (e.g., using interactive software to communicate with peers, exchange ideas, and collaborate) (Troussas et al., 2021a).

Regarding teachers’ emotions (RQ3), a less explored variable in ERT, the data unequivocally showed that teachers experienced fewer positive emotions and more negative emotions, especially among teachers in primary and secondary education, teachers with less than 6 years of experience, women, teachers from public schools and teachers whose students come from low SES. These results are in line with previous research showing teachers’ resilience and level of burnout were significantly correlated with their attitudes toward technology (Sokal et al., 2020), and teachers experiencing strong emotions such as the fear of getting sick or losing their job (Dayal and Tiko, 2020). Furthermore, students from lower SES backgrounds and those whose parents have lower levels of education are statistically less likely to obtain resources from their teachers or to use educational apps (Doyle, 2020).

Finally, when it comes to teachers’ motivation (RQ4), also an aspect not as frequently explored as well-being, our participants’ motivation was low, reporting factors such as worrying about students’ well-being, the impact on their learning process and in the interactions among teachers and students, and how the lockdown was affecting the students and their families. Interestingly, female and higher education teachers reported less motivation, as well as teachers with students in intermediate-low and low SES. Previous research found that the job motivation of university teachers was lower during ERT than before the pandemic, especially for teachers with a negative opinion of university management (Kulikowski et al., 2021b). Higher education teachers’ motivation came from factors that were missing during remote teaching: the perceived relationship with the students and the impact of their instruction on academic development (Han and Yin, 2016; Moorhouse and Kohnke, 2021). In terms of gender, previous studies have tended to indicate higher levels of stress and anxiety in women (Casimiro-Urcos et al., 2020; Hayes et al., 2020; Taylor et al., 2020), which might be connected to their time-consuming activities such as childcare and unpaid domestic labor, among others (Jelińska and Paradowski, 2021).

## Practical Implications and Limitations

Next, we explore the practical implications for the teachers groups we found to be more affected. First, the major detriment to female teachers is a global challenge, which might be related to higher responsibilities in domestic labor and childcare, plus having a higher number of women in primary and secondary education where students were more likely to struggle to keep up with the pace of learning during ERT. Societal interventions are needed to ensure better conditions for female teachers. Second, teachers in public schools and with low SES students have struggled most, probably because of a mix of the students having less experience and access to technological resources plus more constrains and challenges at home, such as shared rooms or lack of internet connection. It is crucial, if we want equalitarian learning opportunities, that we address these deficiencies by investing more resources to offer equal opportunities. Third, our results showed that primary and secondary school teachers were more affected, probably because they work with less mature students and they also have limited experience using online systems in their learning (e.g., learning management systems). The younger the student, the more attention they would require, thus adding workload to teachers who were already struggling with the new situation.

Regarding the limitations of our study, our data come from a survey, which may thus be affected by the usual risks of self-report; however, the variables we explored here are usually measured through self-report, as they evaluate the internal perceptions of the participants. Participation was also voluntary, so our results only report the characteristics of the teachers who felt motivated to participate.

## Conclusion

In conclusion, our study shows that ERT imposed instructional constraints and added pressure on teachers at all levels, decreasing the well-being, positive emotions, and motivation of teachers while increasing negative emotions. Importantly, not all teachers were affected equally, with female teachers, teachers with students from low SES, those teaching in public schools, and primary and secondary teachers as the most affected groups. This indicates that the impact of the switch to ERT differs, and some populations of teachers are more at risk of suffering burnout if ERT continues. The COVID-19 lockdowns have stressed society, and our teachers, as crucial actors, have suffered a considerable impact. We need to provide better solutions if we are to go back to ERT, as some countries are returning to strict lockdowns (e.g., Austria in November 2021). It is our hope that this study can shed light on what areas are most important to address and ways of identifying the most vulnerable teachers.

## Data Availability Statement

The raw data supporting the conclusions of this article will be made available by the authors, without undue reservation.

## Ethics Statement

Ethical review and approval was not required for the study on human participants in accordance with the local legislation and institutional requirements. The patients/participants provided their written informed consent to participate in this study.

## Author Contributions

EP, JF, and LP performed the instrument design and data collection. CR-H performed the data analysis. EP, EB, JF, FD, LP, and CR-H contributed to the literature review and wrote the manuscript, in decreasing order of participation in writing.

## Conflict of Interest

The authors declare that the research was conducted in the absence of any commercial or financial relationships that could be construed as a potential conflict of interest.

## Publisher’s Note

All claims expressed in this article are solely those of the authors and do not necessarily represent those of their affiliated organizations, or those of the publisher, the editors and the reviewers. Any product that may be evaluated in this article, or claim that may be made by its manufacturer, is not guaranteed or endorsed by the publisher.
